# A comparative analysis of hysterectomy outcomes: robotic single-port vs. traditional transvaginal NOTES approaches

**DOI:** 10.3389/fmed.2025.1614384

**Published:** 2025-10-31

**Authors:** Qiannan Yang, Daniel Y. Lovell, Juliana Wu, Chunhua Zhang, Xiaoming Guan

**Affiliations:** ^1^Division of Minimally Invasive Gynecologic Surgery, Baylor College of Medicine, Houston, TX, United States; ^2^John Sealy School of Medicine, University of Texas Medical Branch, Galveston, TX, United States; ^3^Department of Obstetrics and Gynecology, The Second Affiliated Hospital of Nanjing Medical University, Nanjing, Jiangsu, China

**Keywords:** minimal invasive surgery, laparoscopic, robotic single port vNOTES, robotic surgery, transvaginal hysterectomy

## Abstract

**Introduction:**

This study aimed to explore the feasibility and safety of robotic single port vaginal natural orifice transluminal endoscopic surgery (RSP-vNOTES) hysterectomy when compared with traditional vaginal natural orifice transluminal endoscopic surgery (T-vNOTES) hysterectomy.

**Methods:**

In this study, the clinical data of 135 patients who underwent RSP-vNOTES or T-vNOTES hysterectomy performed by a single minimally invasive gynecologic surgeon from January 2017 to September 2024 were retrospectively analyzed. Clinical characteristics, surgical outcomes, perioperative complications, and postoperative pain scores were collected and analyzed.

**Results:**

A total of 79 patients underwent T-vNOTES hysterectomy and 56 patients underwent RSP-vNOTES hysterectomy. Compared to the T-vNOTES group, the RSP-vNOTES group demonstrated a lower median body mass index (27 vs. 30 kg/m^2^, *p* = 0.04), fewer vaginal deliveries (0 vs. 1, *p* = 0.02), and a lower median uterine weight (90 vs. 175 g, *p* = 0.001). In terms of concomitant procedures, the RSP-vNOTES group more frequently underwent interventions related to endometriosis management—including excision of endometriotic lesions, lysis of adhesions, ovarian cystectomy, oophoropexy, bowel shaving, and bowel oversewing (all *p* < 0.05). A multiple linear regression analysis was performed to adjust for these differences. After adjustment, no significant differences were observed between the groups in hysterectomy time, total operative time, estimated blood loss, or postoperative pain scores. Conversion to laparoscopy or laparotomy occurred in six cases in the T-vNOTES group and in one case in the RSP-vNOTES group, although this difference did not reach statistical significance. Similarly, no significant differences were observed in intraoperative or postoperative complications between the groups.

**Discussion:**

When compared to T-vNOTES hysterectomy, RSP-vNOTES hysterectomy appears more feasible and safer for surgery, especially in cases involving concurrent endometriosis resection, and warrants further consideration as a skillset in a gynecologic surgeon’s toolbox. Large multicenter studies involving multiple surgeons and longer follow-up are needed to fully establish the safety and feasibility of this approach.

## 1 Introduction

Hysterectomy is one of the most common gynecologic procedures performed in the United States for a range of benign indications, such as abnormal uterine bleeding, endometriosis, and fibroids (1). Over the past few decades, minimally invasive approaches—whether vaginal or laparoscopic methods—have become preferred due to reduced blood loss, postoperative pain, and infections and faster recovery (2, 3). When possible, vaginal hysterectomy is regarded as the preferred approach according to the American College of Obstetricians and Gynecologists (ACOG) recommendations (4). Compared to abdominal laparoscopic hysterectomies, vaginal hysterectomy has been associated with shorter operative times and decreased costs (5–7). However, rates of vaginal hysterectomy utilization have declined since the advent and growing popularity of the laparoscopic approach, especially among patients with a larger uterine size (8, 9).

However, there has been growing utilization through the transvaginal route with natural orifice transluminal endoscopic surgery (NOTES) to perform gynecologic procedures, including hysterectomy. Traditional vaginal natural orifice transluminal endoscopic surgery (T-vNOTES) has been associated with reduced postoperative pain, decreased risk of complications and risks, and improved cosmetic results—likely due to unnecessary abdominal port incision sites (10). Additionally, vNOTES hysterectomy has been shown to be safe with comparative outcomes to conventional vaginal hysterectomy (11, 12). The limited use of vNOTES for hysterectomy is likely a result of the learning curve and technical challenges with this route (13).

The advent of robotic assistance in the setting of vNOTES may offer a solution (14). Studies have shown robotic multiple-port vNOTES (RMP-vNOTES) to be safe and have comparative outcomes to T-vNOTES, traditional abdominal laparoscopic hysterectomy, and abdominal robotic single-site hysterectomy (15–17). The Da Vinci SP (Intuitive Systems) is a single-port robotic surgery system that uses three double-elbowed instruments and a 3D HD multi-jointed endoscope through a 25-mm site. These features allow for increased motion in confined spaces, which may help lessen the challenges noticed in single-site and natural orifice surgery. FDA approval has been granted for the Da Vinci SP system for certain urologic and otolaryngologic procedures. However, there are limited studies that have evaluated the efficacy of utilizing the Da Vinci SP system for gynecologic procedures. Notably, Guan et al. (18) reported the first case of robotic single port vaginal NOTES (RSP-vNOTES) hysterectomy. A recent comparative study of RSP-vNOTES and RMP-vNOTES demonstrated that both robotic-assisted vNOTES approaches are safe and effective; however, RSP-vNOTES provides logistical and ergonomic advantages and facilitates the management of more complex procedures, particularly endometriosis excision (19). Currently, no studies have directly evaluated the outcomes of RSP-vNOTES vs. T-vNOTES hysterectomy because both approaches utilize the same natural orifice route and transvaginal access, and this comparison allows for a direct assessment of outcomes. This retrospective cohort study was designed to compare surgical outcomes between patients undergoing T-vNOTES and RSP-vNOTES hysterectomy at a single institution, thereby addressing this gap and providing clinically relevant evidence on whether RSP-vNOTES offers advantages in ergonomics, visualization, and the management of complex cases compared with the conventional T-vNOTES platform.

## 2 Materials and methods

A retrospective chart review was performed for all patients undergoing T-vNOTES or RSP-vNOTES hysterectomy between January 2017 and September 2024. All procedures were performed at Baylor College of Medicine hospital affiliates (Baylor St. Luke’s Medical Center and Texas Children’s Pavilion for Women) by one fellowship-trained minimally invasive gynecologic surgeon (X. Guan). This study was approved by the institutional review board at Baylor College of Medicine.

Medical records were identified through the surgeon’s case log during the study period through a secure portal. Demographic data were collected, including age, ethnicity, body mass index (BMI), and history of abdominal or pelvic surgery. The type of procedures, total operative time, conversion to abdominal laparoscopy or laparotomy, estimated blood loss, uterine weight, same-day discharge, and postoperative pain were also obtained. Intra-operative and postoperative complications were noted, with patients followed for 3 to 6 weeks postoperation. From July 2018, patients were asked to complete a questionnaire during their postoperative visits, where they were asked to rate their average pain level at weeks 1, 2, and 3 after surgery.

### 2.1 Surgical technique

Following general endotracheal anesthesia, the patient was positioned and draped in a dorsal lithotomy position with their arms secured to their sides. It is typically our practice to place bilateral temporary ureteral stents with indocyanine green (ICG) injection for patients who undergo RSP-vNOTES at the beginning of the procedure. ICG stent placement has been previously described as a method to potentially decrease operative times and ureteral injury (20). Following stent placement, a Foley catheter is placed for the duration of surgery.

In both T-vNOTES and RSP-vNOTES hysterectomy, the traditional steps of a vaginal hysterectomy were first performed, ideally until bilateral uterine artery pedicles were secured. Once it was no longer feasible to continue the hysterectomy transvaginally, a vaginal port was placed using “4-P” port anchoring methods (21). Limitations to continue the vaginal hysterectomy are typically secondary to adhesions in the vesicouterine or rectovaginal spaces. In these cases, the GelPOINT Mini^®^ advanced access platform (Applied Medical, Rancho Santa Margarita, CA, United States) was used for patients undergoing T-vNOTES, which allows two laparoscopic instruments and the laparoscopic camera. In patients undergoing RSP-vNOTES, the SP access port kit (Intuitive Surgical, Sunnyvale, CA, United States) was used, followed by docking of the Da Vinci Single Port robotic system (Intuitive Surgical, Sunnyvale). The SP platform accommodates three instruments and one robotic camera.

The remaining steps of the hysterectomy were then completed, followed by any additional indicated procedures such as excision of endometriosis, oophorectomy, oophoropexy, ovarian cystectomy, or high uterosacral ligament suspension. After the completion of surgery, the GelPOINT Mini platform or the SP access port kit was removed, and the vaginal cuff was sewn vaginally.

### 2.2 Data analysis

All continuous variables were tested for normality using descriptive statistics for skewness and kurtosis, visual evaluation of histograms, and the Kolmogorov–Smirnov test. As our primary outcomes did not have a normal distribution, all continuous data were described as median [interquartile range (IQR)] to maintain consistency, and group differences were evaluated using the Mann–Whitney *U*-test. Categorical variables were expressed as percentages and analyzed using Fisher’s exact test or Pearson’s chi-square test, as appropriate. To account for potential confounding factors such as age, uterine weight, vaginal deliveries, and additional procedures, a multivariable linear regression analysis was performed for surgical outcomes. All statistical analyses were carried out using SPSS software (version 25.0; SPSS Inc., Chicago, IL, United States), and the level of significance was set at a *p*-value of <0.05.

## 3 Results

A total of 135 patients underwent T-vNOTES hysterectomy (79 patients from January 2017 to August 2020) or RSP-vNOTES hysterectomy (56 patients from November 2023 to September 2024).

### 3.1 Patient characteristics

Patient characteristics such as age, BMI, ethnicity, and number of previous vaginal deliveries differed statistically between the T-vNOTES and RSP-vNOTES hysterectomy groups. The median age for subjects in the T-vNOTES group was 44 years, compared with a median age of 39 years in the RSP-vNOTES group (*p* = 0.001). Compared to the T-vNOTES group, the BMI in the RSP-vNOTES group was slightly lower (30 [25–34] kg/m^2^ vs. 27 [23–30] kg/m^2^, *p* = 0.04). In addition, the number of previous vaginal deliveries between the T-vNOTES and RSP-vNOTES groups was 1 [0–2] vs. 0 [0–1] (*p* = 0.02). The number of previous abdominal or cervical surgeries did not differ between the two groups. Table 1 details the characteristics of the study participants in both groups.

**Table 1:** Characteristics of the study participants in the two study groups (*N* = 135).

Characteristic	T-vNOTES (*N* = 79)	RSP-vNOTES (*N* = 56)	Difference/OR (95% CI)	*r/χ^2^* value	*P-*value
Age, years	44 [39–49]	39 [34–43]	−6 [−9, −4]	*r* = −0.46	0.001
Body mass index, kg/m^2^	30 [25–34]	27 [23–30]	−2 [−4, 0]	*r* = −0.21	0.04
Ethnicity				*χ^2^* = 24.1	0.001
Caucasian	27 (34.2)	43 (76.8)			
African American	27 (34.2)	6 (10.7)			
Asian	12 (15.2)	4 (7.1)			
Hispanic	13 (16.5)	3 (5.4)			
Tobacco use	5 (6.3)	3 (5.4)	0.83 [0.19, 3.54]	Fisher’s	1.00
Number of vaginal deliveries	1 [0–2]	0 [0–1]	0 [−1, 0]	*r* = −0.21	0.02
Number of previous cervical procedures	0 [0–0]	0 [0–0]	0 [0, 0]	*r* = −0.03	0.57
Number of previous abdominal surgeries	1 [0–3]	1 [0–3]	0 [0, 1]	*r* = −0.09	0.35

Values are presented as median [interquartile range] for continuous variables and *N* (%) for categorical variables. Differences between groups were assessed using the Mann–Whitney *U* test for continuous variables, reported as median difference (Hodges–Lehmann estimate with 95% confidence interval [CI]) and effect size (rank-biserial correlation, *r*), and using Pearson’s chi-squared test or Fisher’s exact test for categorical variables, reported as odds ratio (OR) with 95% CI and *χ*^2^ values as appropriate.

T-vNOTES, traditional vaginal natural orifice transluminal endoscopic surgery; RSP-vNOTES, robotic single-port vaginal natural orifice transluminal endoscopic surgery; CI, confidence interval; OR, odds ratio.

### 3.2 Primary indications

**Figure 1:**
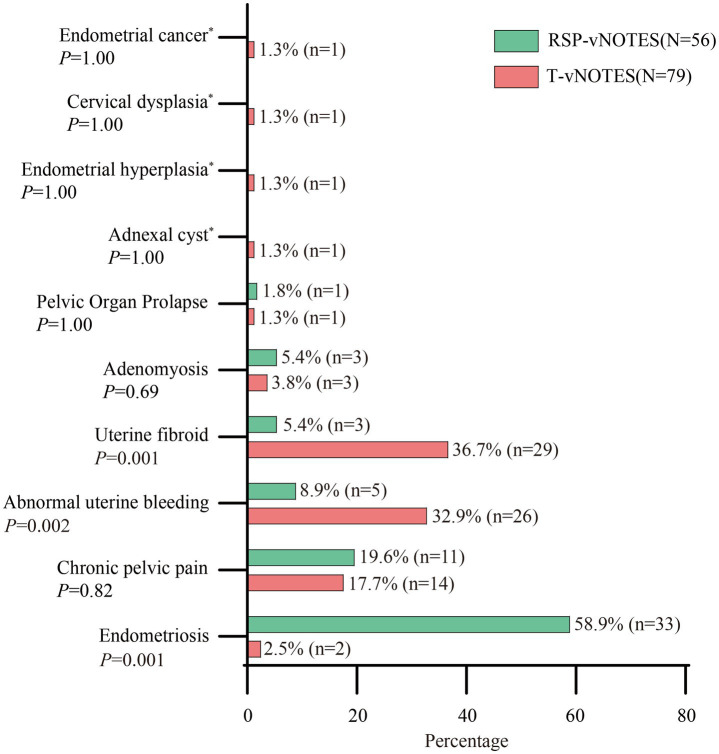
Distribution of primary indications in the two study groups. T-vNOTES, traditional vaginal natural orifice transluminal endoscopic surgery; RSP-vNOTES, robotic single port vaginal natural orifice transluminal endoscopic surgery. * means 0 patient in the RSP-vNOTES group.

The primary indications for surgery, as shown in Figure 1, included endometriosis, chronic pelvic pain, abnormal uterine bleeding (AUB), uterine fibroids, and pelvic organ prolapse in both groups. In the T-vNOTES cohort, other indications included adnexal cyst (1.3%), endometrial hyperplasia (1.3%), cervical dysplasia (1.3%), and endometrial cancer (1.3%). The RSP-vNOTES group had a predominance of endometriosis (*p* = 0.001), with fewer cases of AUB (*p* = 0.002) and uterine fibroid (*p* = 0.001) compared to the T-vNOTES group.

### 3.3 Additional surgical procedures at time of hysterectomy

**Figure 2:**
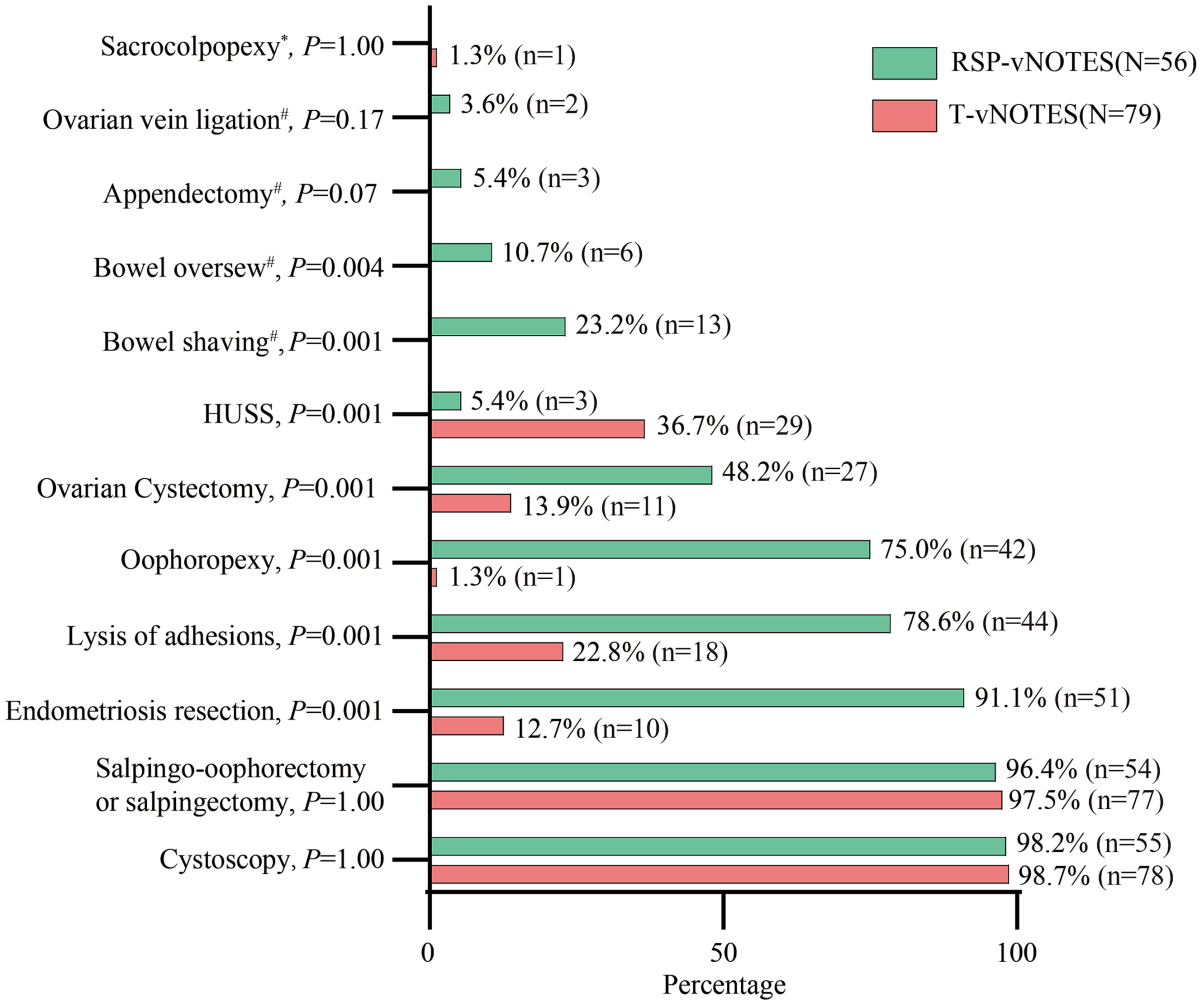
The additional surgical procedures performed. T-vNOTES, traditional vaginal natural orifice transluminal endoscopic surgery; RSP-vNOTES, robotic single port vaginal natural orifice transluminal endoscopic surgery; HUSS, high uterosacral ligament suspension. * means 0 patient in the RSP-vNOTES group, # means 0 patient in the T-vNOTES group.

The type and breakdown of additional surgical procedures are outlined in Figure 2. Endometriosis excision, lysis of adhesions, bowel shaving, bowel repair, oophoropexy, and ovarian cystectomy were more frequently performed at the time of RSP-vNOTES hysterectomy (all *p* < 0.05). High uterosacral ligament suspension (HUSS) procedures were more frequently performed at the time of T-vNOTES (*p* = 0.001).

### 3.4 Intraoperative and postoperative surgical outcomes

Table 2 provides a summary of the surgical outcomes. Hysterectomy time did not differ between the groups. Total operative time was longer in the RSP-vNOTES group than in the T-vNOTES group (157 [142–194] vs. 143 [114–181] min, *p* = 0.01). Notably, estimated blood loss was lower in the RSP-vNOTES group (25 [25–50] vs. 50 [25–150] mL, *p* = 0.001). Based on the pathology report, uterine weight was significantly lower in the RSP-vNOTES group (90 [58–136] vs. 175 [92–393], *p* = 0.001). Other surgical outcomes, such as the rate of same-day discharge and conversion, were not significantly different. Same-day discharge was defined as patients being discharged within 24 h after surgery.

**Table 2:** Surgical outcomes between the two study groups (*N* = 135).

Variables	T-vNOTES (*N* = 79)	RSP-vNOTES (*N* = 56)	Difference/OR (95% CI)	*r/χ^2^* value	*P-*value
Uterine weight, g	175 [92–393]	90 [58–136]	−68 [−119, −31]	*r* = −0.39	0.001
Hysterectomy time, min	44 [31–58]	42 [30–56]	−2 [−9, 5]	*r* = −0.05	0.60
Total operative time, min	143 [114–181]	157 [142–194]	20 [4, 34]	*r* = −0.25	0.01
Estimated blood loss, mL	50 [25–150]	25 [25–50]	−25 [−50, −5]	*r* = −0.42	0.001
Same-day discharge	73 (92.4)	51 (91.1)	0.84 [0.24, 2.97]	Fisher’s	0.76
Conversion	6(7.6)	1 (1.8)	0.22 [0.03, 1.72]	Fisher’s	0.24
Pain score, week 1	6 [3–8]	7 [4–9]	1 [0, 2]	*r* = −0.22	0.09
Pain score, week 2	4 [0–6]	5 [2–7]	1 [0, 3]	*r* = −0.23	0.07
Pain score, week 3	1 [0–4]	4 [1–5]	1 [0, 3]	*r* = −0.28	0.03

Values are presented as median [interquartile range] for continuous variables and *N* (%) for categorical variables. Differences between groups were assessed using the Mann–Whitney *U* test for continuous variables, reported as median difference (Hodges–Lehmann estimate with 95% confidence interval [CI]) and effect size (rank-biserial correlation, *r*), and using Pearson’s chi-squared test or Fisher’s exact test for categorical variables, reported as odds ratio (OR) with 95% CI and *χ*^2^ values as appropriate.

Since July 2018, we have implemented a postoperative pain questionnaire into clinical practice. Therefore, pain scale data were available for only 34 patients in the T-vNOTES group and 52 patients in the RSP-vNOTES group. There were no significant differences in pain scores at weeks 1 and 2; however, pain levels at week 3 were higher in the RSP-vNOTES group (4 [1–5] vs. 1 [0–4], *p* = 0.03).

Because age, BMI, number of vaginal deliveries, uterine weight, and additional procedures (including endometriosis excision, lysis of adhesions, bowel shaving, bowel repair, oophoropexy, ovarian cystectomy, and HUSS) differed between the two study groups, adjustments were made for these variables. After adjustment, differences in total operative time, estimated blood loss, and postoperative pain at week 3 were no longer statistically significant (Table 3).

**Table 3:** Multivariable linear regression results.

Outcome	*Β* coefficient	*p-*value	95% CI for B	
			Lower bound	Upper bound
Hysterectomy time, min	16.962	0.076	−1.815	35.739
Total operative time, min	4.012	0.821	−30.984	39.009
Estimated blood loss, mL	−84.874	0.054	−171.320	1.572
Pain score, week 1	0.745	0.585	−1.962	3.452
Pain score, week 2	0.950	0.527	−2.028	3.927
Pain score, week 3	1.270	0.304	−1.174	3.714

Adjustments were made for variables age, body mass index, vaginal deliveries, uterine weight, and additional procedures (endometriosis excision, lysis of adhesions, bowel shaving, bowel oversew, oophoropexy, ovarian cystectomy, and high uterosacral suspension).

The results are presented as regression coefficients (*B*) with corresponding 95% confidence intervals (CI) and *p*-values.

The total complication rate was 20.3% (16 cases) in the T-vNOTES group and 19.6% (11 cases) in the RSP-vNOTES group, with no statistically significant difference between the two groups (Table 4). Postoperative complications were classified according to the Clavien–Dindo (CD) system. In the T-vNOTES group, 2 of 79 patients (2.5%) experienced intraoperative complications, and 14 patients developed postoperative complications, including 11.4% with CD grade I–II and 6.3% with CD grade III–IV complications. In the RSP-vNOTES group, no intraoperative complications occurred, and all 11 complications were postoperative, comprising 10.7% with CD grade I–II and 8.9% with CD grade III complications; no CD grade IV events were observed.

**Table 4:** Intraoperative and postoperative complications in the two study groups (*N* = 135).

Complications	T-vNOTES (N = 79)	RSP-vNOTES (N = 56)	OR (95% CI)	*χ^2^* value	*P-*value
Total complications	16 (20.3)	11 (19.6)	0.96 [0.41, 2.26]	0.008	0.93
Intraoperative complications	2 (2.5)	0			
Bladder injury	1 (1.3)	0			
Blood transfusion	1 (1.3)	0			
CD grade I-II	9 (11.4)	6 (10.7)	1.07 [0.36, 3.20]	0.015	0.90
Urinary retention	2 (2.5)	0			
Pain	2 (2.5)	0			
Urinary tract infection	4 (5.1)	5 (8.9)			
Pneumonia	1 (1.3)	0			
Vaginal cuff infection	0	1 (1.8)			
CD grade III (reoperation)-IVa	5 (6.3)	5 (8.9)	1.45 [0.40, 5.27]	Fisher’s	0.74
Vaginal cuff reoperation	2 (2.5)	2 (3.6)			
Pelvic abscess/fluid collection drainage	1 (1.3)	2 (3.6)			
Persistent pain	1 (1.3)	0			
Abdominal hematoma	0	1 (1.8)			
Stroke	1 (1.3)	0			

Values are presented as *N* (%) for categorical variables. Differences between groups were assessed using Pearson’s chi-squared test or Fisher’s exact test, reported as odds ratio (OR) with 95% CI and *χ*^2^ values as appropriate.

T-vNOTES, traditional vaginal natural orifice transluminal endoscopic surgery; RSP-vNOTES, robotic single-port vaginal natural orifice transluminal endoscopic surgery; CI, confidence interval; OR, odds ratio.

## 4 Discussion

To the best of our knowledge, this is the first comparative study between patients undergoing T-vNOTES hysterectomy and RSP-vNOTES hysterectomy for gynecologic disease. T-vNOTES offers safer and less invasive techniques while addressing the challenges of exposure associated with traditional transvaginal surgery (22). Nevertheless, there are still several limitations with T-vNOTES, especially with regard to surgical instrument interference and the limitations in surgical triangulation (17). Compared to T-vNOTES, RMP-vNOTES has significant advantages. The three-dimensional high-definition imaging improves the surgical field of view. Wristed robotic instruments enable 360° exploration and facilitate complex procedures, including anastomosis and suture knot tying. The robotic platform also eliminates tremors, improves surgical accuracy, and reduces the risk of nerve and vascular damage (23). However, RMP-vNOTES also has certain limitations, including the use of only two robotic arms, a straight camera, and ergonomic challenges associated with single-port surgery. Therefore, RMP-vNOTES is considered a transitional procedure platform to RSP-vNOTES. The development and implementation of a unique single-port robotic platform in minimally invasive gynecologic procedures, notably in the context of vNOTES, marks a significant step forward in surgical technology. Prior to our investigation, there has been limited data published regarding the safety and feasibility of RSP-vNOTES hysterectomy. Few studies currently exist describing the use of RSP-vNOTES in gynecologic procedures. These studies utilize either the Da Vinci SP robotic system for hysterectomy or the Chinese robotic single-port platform (EDGE SP 1000, Jingfeng, Shenzhen, China) for hysterectomy, ovarian cystectomy, and sacrocolpopexy, demonstrating promising surgical outcomes (18, 19, 24–27).

Although the total operative time was significantly different between the two groups, the hysterectomy time was not. Compared to the T-vNOTES group, the median total operative time was longer in the RSP-vNOTES group (143 vs. 157 min). The median operative time of RA-vNOTES hysterectomy without endometriosis resection, reported by previous studies, varied from 108 to 156 min (14, 16, 28). Xu et al. (29) published an article showing that the mean operative time of RA-vNOTES hysterectomy with stage IV endometriosis resection was 224.3 min. The above data indicate that, although the total operative time in the RSP-vNOTES group is longer than that in the T-vNOTES group, it is consistent with previously reported robotic surgery times. The longer operative time in the RSP-vNOTES group was primarily attributable to the greater number of additional surgical procedures performed and not to the use of the SP robotic platform itself. Most of the patients underwent endometriosis resection, lysis of adhesions, oophoropexy, ovarian cystectomy, bowel shaving, and bowel oversewing for the treatment of endometriosis at the time of RSP-vNOTES hysterectomy. The higher number of patients receiving endometriosis treatment in the RSP-vNOTES group was due to differences in primary indications between the two groups. In the RSP-vNOTES group, the indication for 44 of 56 patients (78.5%) was endometriosis (58.9%) or chronic pelvic pain (19.6%). In contrast, the main indications in the T-vNOTES group were uterine fibroid (36.7%) and abnormal uterine bleeding (32.9%). This suggests that robotic surgery offers additional benefits, such as improved dissection precision around vasculature, bowels, and ureters, which are areas commonly involved in endometriosis resection.

When considering other surgical outcomes between the two study groups, there was no difference in estimated blood loss, same-day discharge rates, number of conversions, or postoperative pain scores after adjustment. A total of six patients in the T-vNOTES group underwent conversion to laparoscopic or laparotomy surgeries. One patient had a successful T-vNOTES hysterectomy; however, the uterus could not be placed in a bag vaginally because of its large size, requiring a mini-laparotomy for removal of the uterus. Two patients were converted to traditional three-port total laparoscopic hysterectomy: one due to limited visualization and failure to achieve vaginal insufflation or to perform colpotomy and the other due to bleeding from the right uterine artery pedicle with failure to achieve hemostasis. The remaining three patients were converted to single-incision total laparoscopic hysterectomy: two due to a large broad ligament fibroid and an anterior myoma preventing safe colpotomy and one with stage IV endometriosis and complete posterior cul-de-sac obliteration, which made colpotomy impossible. Only one patient in the RSP-vNOTES group required a mini-laparotomy to remove the uterus after the completion of the robotic transvaginal NOTES procedures. Despite the irregularly shaped, large uterus, the robotic transvaginal NOTES procedure was completed within 3 h. However, difficulty arose during placement of the uterus into the containment bag for tissue extraction due to an insufflation malfunction and the challenge of finding a bag large enough to accommodate the specimen. In conclusion, the majority of these conversions occurred early in the primary surgeon’s T-vNOTES or RSP-vNOTES experience. With greater surgeon experience, it is probable that these conversions would not have been necessary.

Two patients had intraoperative complications: one bladder injury (1 of 135 = 0.7%) and one intraoperative blood transfusion (1 of 135 = 0.7%). As for the postoperative complications in the two study groups, urinary tract infection was the most common postoperative complication, with a total of 9 of 135 (6.7%) patients diagnosed. Two other patients had immediate postoperative urinary retention and were discharged home with a Foley catheter placement. Both successfully passed a voiding trial within 1 week of surgery. One patient had a vaginal cuff infection and was treated with oral antibiotics. Four patients underwent a vaginal cuff procedure due to vaginal cuff bleeding or an abscess. Three patients had drainage by interventional radiology for a pelvic abscess or pelvic fluid collection. One patient had a laparoscopic procedure for an abdominal hematoma. One patient undergoing reoperation had persistent postoperative pain 3 weeks after T-vNOTES hysterectomy with bilateral salpingectomy, which she believed was because her ovaries were left *in situ*, and after adequate counseling of risks and benefits, she elected to proceed with bilateral oophorectomy in a shared decision-making model. The most serious complication occurred in a patient with multiple medical comorbidities, who was readmitted on postoperative day 24 with a left pontine stroke.

Robotic-assisted approaches, including RSP-vNOTES, offer enhanced visualization and instrument dexterity that are particularly advantageous in the management of advanced endometriosis. Our findings are consistent with the growing role of robotic-assisted gynecologic surgery in this setting, as highlighted by recent studies (30), and this study represents an early contribution to that evolving paradigm. While RSP-vNOTES demonstrates promising clinical utility, its adoption remains constrained by technical challenges, a distinct learning curve, and higher costs, making it currently most applicable to specialized centers with the requisite expertise and resources. In clinical practice, a potential framework may involve reserving classical vaginal hysterectomy for simple cases (often with concomitant prolapse surgery), utilizing vNOTES for more complex hysterectomy and adnexal procedures, and considering robotic SP-vNOTES as a high-end option for deep infiltrating endometriosis and selected oncologic indications, while acknowledging that the latter is presently most feasible in highly specialized centers.

Despite our findings, our study has some weaknesses. First, as all procedures were performed by a single minimally invasive surgeon with substantial experience in both T-vNOTES and RSP-vNOTES, the external validity of our findings may be limited, particularly for surgeons with different levels of expertise or institutions with varying practice environments. Second, the use of retrospective and non-contemporaneous cohorts may introduce recall and selection biases, including demographic and clinical heterogeneity, the effects of evolving surgical expertise, and changing patient selection criteria over time, which represent important limitations of our study. However, the two surgical techniques remain comparable, as both require a surgeon to overcome a learning curve. Although the surgeon had extensive prior experience with vNOTES when initiating RSP-vNOTES procedures, the operative techniques of RSP-vNOTES differ substantially from those of conventional vNOTES and the RMP-vNOTES platform, necessitating additional time for mastery. First, the curved-wristed instruments used in the single-port platform are markedly different from the straight instruments of the RMP system, requiring the surgeon to adapt to altered tactile feedback and force modulation. Second, the SP platform’s unique, flexible, multi-jointed endoscope requires additional training to achieve optimal positioning and maintain stable visualization within the confined transvaginal working space. Therefore, both groups in our study reflect the initial learning and adaptation phases of their respective techniques, supporting their comparability. Third, we implemented the postoperative pain questionnaire into practice in July 2018, which excluded nearly half of the patients in the T-vNOTES group from the pain score statistical analysis. Overall, we still believe that this study yields useful information for further advancement in the use of vNOTES on the robotic single-port platform. Future research should incorporate multicenter studies involving multiple surgeons to validate our findings across more diverse patient populations and surgical settings.

## 5 Conclusion

In conclusion, RSP-vNOTES appears as a feasible option for complex surgeries requiring bilateral pelvic dissection or suturing, including all stages of endometriosis, thereby expanding the surgical indications of vNOTES compared to T-vNOTES. Future multicenter studies with longer follow-up and randomized comparisons to other minimally invasive approaches are needed to fully establish the safety and efficacy of RSP-vNOTES hysterectomy.

## Data Availability

The original contributions presented in the study are included in the article/supplementary material, and further inquiries can be directed to the corresponding authors.
